# Unravelling polar lipids dynamics during embryonic development of two sympatric brachyuran crabs (*Carcinus maenas* and *Necora puber*) using lipidomics

**DOI:** 10.1038/srep14549

**Published:** 2015-09-30

**Authors:** Felisa Rey, Eliana Alves, Tânia Melo, Pedro Domingues, Henrique Queiroga, Rui Rosa, M. Rosário M. Domingues, Ricardo Calado

**Affiliations:** 1 Departamento de Biologia & CESAM, Universidade de Aveiro, Campus Universitário de Santiago, 3810-193 Aveiro, Portugal; 2 Mass Spectrometry Centre, Department of Chemistry & QOPNA, Universidade de Aveiro, Campus Universitário de Santiago, 3810‐193 Aveiro, Portugal; 3 MARE – Marine and Environmental Sciences Centre, Laboratório Marítimo da Guia, Faculdade de Ciências da Universidade de Lisboa, Campo Grande, 1749-016 Lisboa, Portugal

## Abstract

Embryogenesis is an important stage of marine invertebrates with bi-phasic life cycles, as it conditions their larval and adult life. Throughout embryogenesis, phospholipids (PL) play a key role as an energy source, as well as constituents of biological membranes. However, the dynamics of PL during embryogenesis in marine invertebrates is still poorly studied. The present work used a lipidomic approach to determine how polar lipid profiles shift during embryogenesis in two sympatric estuarine crabs, *Carcinus maenas* and *Necora puber*. The combination of thin layer chromatography, liquid chromatography – mass spectrometry and gas chromatography – mass spectrometry allowed us to achieve an unprecedented resolution on PL classes and molecular species present on newly extruded embryos (stage 1) and those near hatching (stage 3). Embryogenesis proved to be a dynamic process, with four PL classes being recorded in stage 1 embryos (68 molecular species in total) and seven PL classes at stage 3 embryos (98 molecular species in total). The low interspecific difference recorded in the lipidomic profiles of stage 1 embryos appears to indicate the existence of similar maternal investment. The same pattern was recorded for stage 3 embryos revealing a similar catabolism of embryonic resources during incubation for both crab species.

The European green crab *Carcinus maenas* and the velvet swimming crab *Necora puber* are two sympatric brachyuran crabs that commonly occur in estuarine habitats of Western Europe^1^. *Carcinus maenas* is a keystone species commonly used as a model in ecological studies, whereas *N. puber* is a commercially important species for coastal fisheries. Brachyuran crabs commonly display a bi-phasic life cycle, with a pelagic larval phase developing in open ocean waters and a benthic post-larval phase that occurs in coastal and estuarine habitats^2^. Decapod embryos are incubated in the abdomen of females (with the exception of penaeiod shrimps); being lecithotrophic throughout their embryonic development, they solely rely on the catabolism of yolk reserves originating from maternal investment for energy and organogenesis^3^.

Lipid reserves catabolism is the main energetic pathway that fuels embryonic development in decapod crustaceans^4^. However, most studies performed so far on crustacean embryos solely focus their fatty acid (FA) profile^5^ and commonly overlook polar lipids. Phospholipids (PL) are important polar lipids being the major constituent of biological membranes and involved in a range of cellular functions (*e.g*., stabilization of proteins within the membrane, protein folding and cofactors in enzymatic reactions)^6^. Moreover, they are also precursors of biologically active mediators which play important functions at metabolic and physiologic levels (*e.g*., eicosanoids, diacylglycerols, inositol phosphates)^7^. PL are also essential for the absorption, transport and storage of lipids, acting as a rich source of essential FAs (EFAs) more than neutral lipids such as triacylglycerols (TAG)^8^.

In the present study we used a lipidomic approach, combining the use of thin layer chromatography (TLC), liquid chromatography – mass spectrometry (LC-MS/MS) and gas chromatography – mass spectrometry (GC-MS), to analyse the PL profile of *C. maenas* and *N. puber* during embryogenesis. We compared resource partitioning and maternal investment between these two sympatric and phylogenetically close species (both are members of family Portunidae^9^) by analysing newly extruded embryos. At this stage, lipid profiles closely reflect maternal diets and can be considered a reliable proxy of quantitative and qualitative maternal investment^10^,^11^. Additionally, we also analysed embryos close to hatching in order to unravel any interspecific differences in lipid dynamics during embryogenesis, with emphasis to PL classes and their molecular species.

## Results

The limited number of samples processed per crab species and embryonic stage (n=3) appeared to have no influence on our results given the little variation recorded among replicates. All significant differences recorded on the relative content of each PL class, molecular species and FA are highlighted in the figures presented, along with accurate *P* values for each statistical test performed.

### Identification of PL classes and quantification by TLC

**Figure 1:**
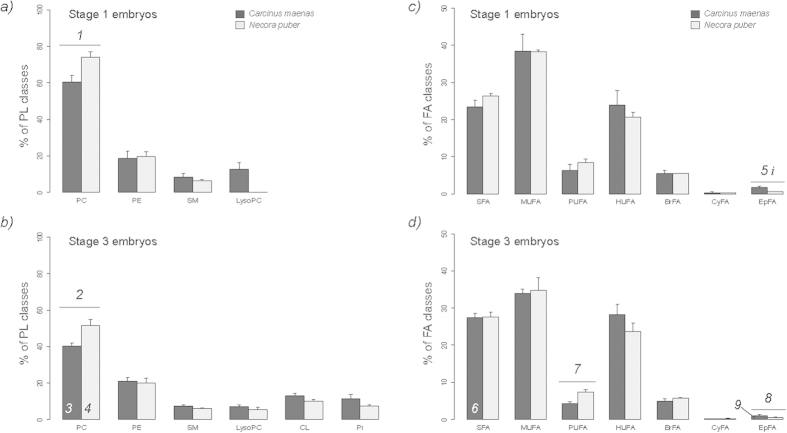
(**a**) Relative abundance of phospholipids classes separated by thin layer chromatography in embryos at stage 1 and (**b**) at stage 3, and (**c**) fatty acid (FA) class profiles in embryos at stage 1 and (**d**) stage 3 of *Carcinus maenas* and *Necora puber*. Error bars represent standard deviation of three independent samples. *P* values for each significant statistical test performed are represented in the figure with a number, with significant differences between crab species being represented on the top of the graph bars and significant differences between stages of the same crab species being represented within the bar of stage 3 embryos. Significant interaction between species and stage is represented with an *i* on the top of stage 1 bars. (Post hoc Tukey HSD, *1*: *P *= 0.0027; *2*: *P *= 0.0095; *3*: *P *= 0.0002; *4*: *P *= 0.0001; *5*: *P *= 0.0001; *6*: *P *= 0.0266; *7*: *P *= 0.0175; *8*: *P *= 0.0361; *9*: *P *= 0.0050). Interaction species *vs* stage (*i*): Epoxy FA (*P *= 0.0081). Abbreviations: PC - phosphatidylcholine; PE - phosphatidylethanolamine; SM - sphingomyelin; LysoPC - Lysophosphatidylcholine; CL - cardiolipin; PI - phosphatidylinositol SFA – Saturated FA; MUFA – Monounsaturated FA; PUFA – Polyunsaturated FA; HUFA – Highly-polyunsaturated FA; BrFA – Branched FA; CyFA – Cyclic FA; EpFA – Epoxy FA.

The fractioning of total lipid extracts in TLC plates showed two different profiles for each stage of embryogenesis (Fig. 1a,b). The separation by TLC enabled to identify phosphatidylcholine (PC), phosphatidylethanolamine (PE) and sphingomyelin (SM) as the PL classes present in the initial stage of embryogenesis (Fig. 1a). LysoPC was observed at stage 1 embryos of *C. maenas*, but not on *N. puber*. At the end of embryonic development (stage 3) the separation by TLC revealed two new classes: phosphatidylinositol (PI) and cardiolipin (CL) (Fig. 1b). In both crab species and stages, the most abundant PL classes were PCs (*C. maenas*: 60.76 ± 4.28% and 40.15 ± 1.83%; *N. puber*: 74.13 ± 2.74% and 51.40 ± 3.58%, at stage 1 and 3, respectively) and PEs (*C. maenas*: 21.61 ± 1.33% and 20.89 ± 2.02%; *N. puber*: 19.63 ± 2.62% and 19.90 ± 2.94%, at stage 1 and 3, respectively).

### Identification of molecular profile in PL classes by hydrophilic interaction liquid chromatography mass spectrometry (HILIC-ESI-MS)

In general, the HILIC-ESI-MS analysis confirmed the lipidomic profile revealed by TLC. However, this technique allowed to identify a new PL class in the embryos of *N. puber* at stage 1 which had not been previously detected by TLC: lysophosphatidylethanolamine (LysoPE) (see supplementary Fig. S1). A total of 98 molecular species were identified in the seven PL classes detected. In the next sections, the composition of the most abundant molecular species, namely the assignment of polar head groups and fatty acyl chain composition (determined by MS/MS^12^), is explained in detail. MS/MS spectra of each PL class can be seen in supplementary Fig. S2 for *C. maenas* and Fig. S3 for *N. puber*. The number of carbon atoms (C) and double bonds (N) (C:N) are described for all molecular species recorded, as well as the FA side chains of the diacyl, plasmanyl or plasmenyl species (whenever possible) (total molecular species identified are listed in supplementary Table S4).

### Phosphatidylcholine (PC) and Lysophosphatidylcholine (LysoPC)

**Figure 2:**
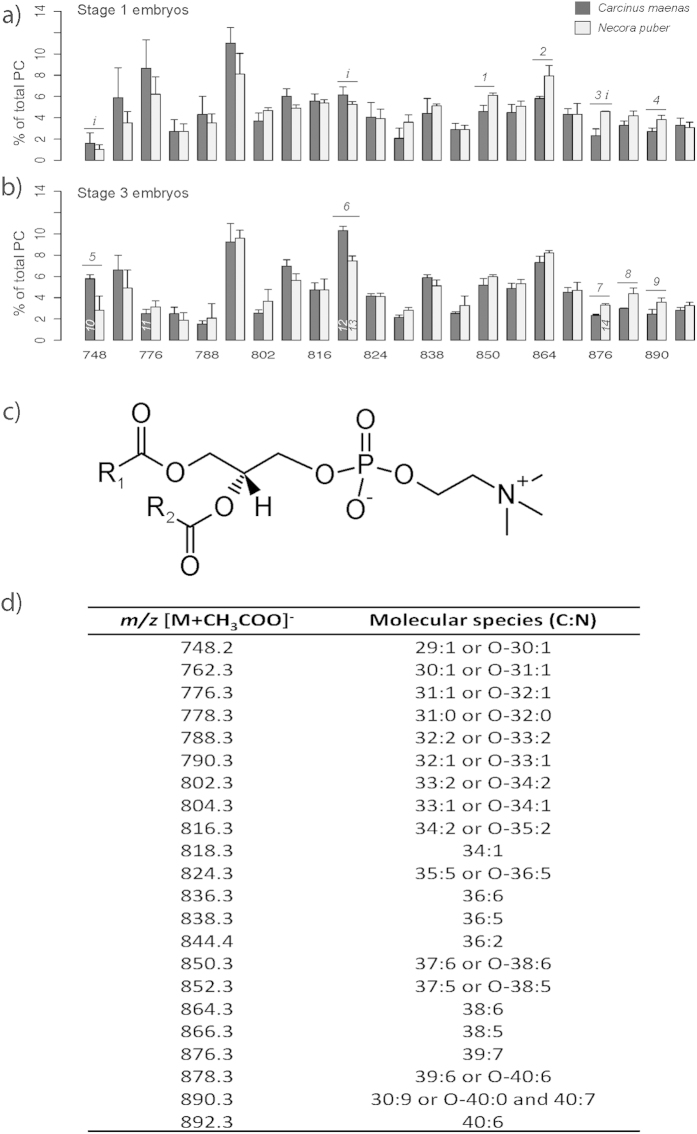
(**a**) Relative abundance of the [M+CH_3_COO]^−^ ions of the different molecular species of phosphatidylcholine (PC) present in the total lipid extract from embryos of *Carcinus maenas* and *Necora puber* at stage 1 and (**b**) stage 3. (**c)** General structure of PC. (**d**) Major molecular species of PC identified by liquid chromatography – mass spectrometry in negative-ion mode in the embryos of *C. maenas* and *N. puber*. Error bars represent standard deviation of three independent samples. *P* values for each significant statistical test performed are represented in the figure with a number, with significant differences between crab species being represented on the top of the graph bars and significant differences between stages of the same crab species being represented within the bar of stage 3 embryos. Significant interaction between species and stage is represented with an *i* on the top of stage 1 bars. (Post hoc Tukey HSD, *1*: *P *= 0.0130; *2*: *P *= 0.0106; *3*: *P *= 0.0002; *4*: *P *= 0.0352; *5*: *P *= 0.0133; *6*: *P *= 0.0005; *7*: *P *= 0.0357; *8*: *P *= 0.0103; *9*: *P* =0.0359; *10*: *P *= 0.0016; *11*: *P *= 0.0066; *12*: *P *= 2.98 × 10^−5^; *13*: *P *= 0.0025; *14*: *P *= 0.0071). Interaction species *vs* stage (*i*): *m/z* 748.2 (*P *= 0.0470); *m/z* 818.3 (*P *= 0.0089); *m/z* 876.3 (*P *= 0.0104).

PCs (Fig. 2c) were the most abundant PL class in both species and stages. Analysis of HILIC-ESI-MS spectra allowed the identification of 22 molecular species on this class and the determination of their molecular composition (C:N) (Fig. 2d). In *C. maenas*, the most abundant species at stage 1 were observed at *m/z* 790.3 and *m/z* 776.3 (Fig. 2a), and identified as PC(32:1) or PC(O-33:1) and PC(31:1) or PC(O-32:1), respectively. Similarly, the most abundant molecular species in *N. puber* were seen at *m/z* 790.3 and *m/z* 864.3, with a possible composition of PC(32:1) or PC(O-33:1) and PC(38:6), respectively (Fig. 2a). At stage 3, the most abundant species were *m/z* 818.3, PC(34:1), and *m/z* 790.3 for *C. maenas* and *m/z* 790.3 and *m/z* 864.3 for *N. puber* (Fig. 2b).

**Figure 3:**
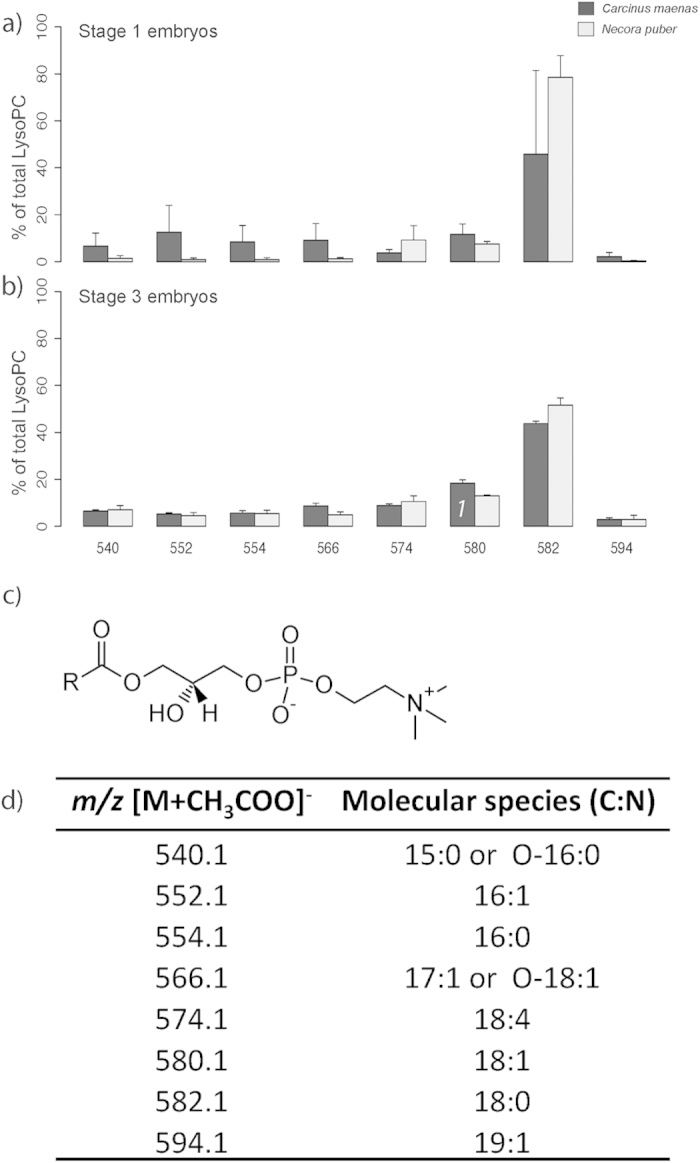
(**a**) Relative abundance of the [M+CH_3_COO]^−^ ions of the different molecular species of lysophosphatidylcholine (LysoPC) present in embryos of *Carcinus maenas* and *Necora puber* at stage 1 and (**b**) at stage 3. (**c**) General structure of LysoPC. (**d**) Major molecular species of LysoPC identified by liquid chromatography – mass spectrometry in negative-ion mode in the embryos of *C. maenas* and *N. puber*. Error bars represent standard deviation of three independent samples. *P* values for each significant statistical test performed are represented in the figure with a number, significant differences between stages of the same crab species being represented within the bar of stage 3 embryos. (Post hoc Tukey HSD, *1*: *P *= 0.0354).

The higher sensitivity of HILIC-ESI-MS, in comparison to TLC, allowed the identification of LysoPCs (Fig. 3c) also in stage 1 embryos of *N. puber*, (Fig. 3a). A total of 8 molecular species were identified by MS/MS (Fig. 3d). At stage 1, the most abundant molecular species in *C. maenas* were at *m/z* 582.1, LysoPC(18:0), and at *m/z* 552.1, LysoPC(16:1), while in *N. puber* were at *m/z* 582.1 and *m/z* 574.1, LysoPC(18:4), (Fig. 3a). At stage 3 embryos the most abundant molecular species for both crab species were observed at *m/z* 582.1 and *m/z* 580.1, LysoPC(18:1), (Fig. 3b). At both embryonic stages the molecular composition of LysoPCs exhibited FAs from 15:0 to 19:1, including two possible LysoPC plasmanyl/plasmenyl species: LysoPC(O-16:0) and LysoPC(O-18:1) (Fig. 3d).

### Phosphatidylethanolamine (PE) and Lysophosphatidylethanolamine (LysoPE)

**Figure 4:**
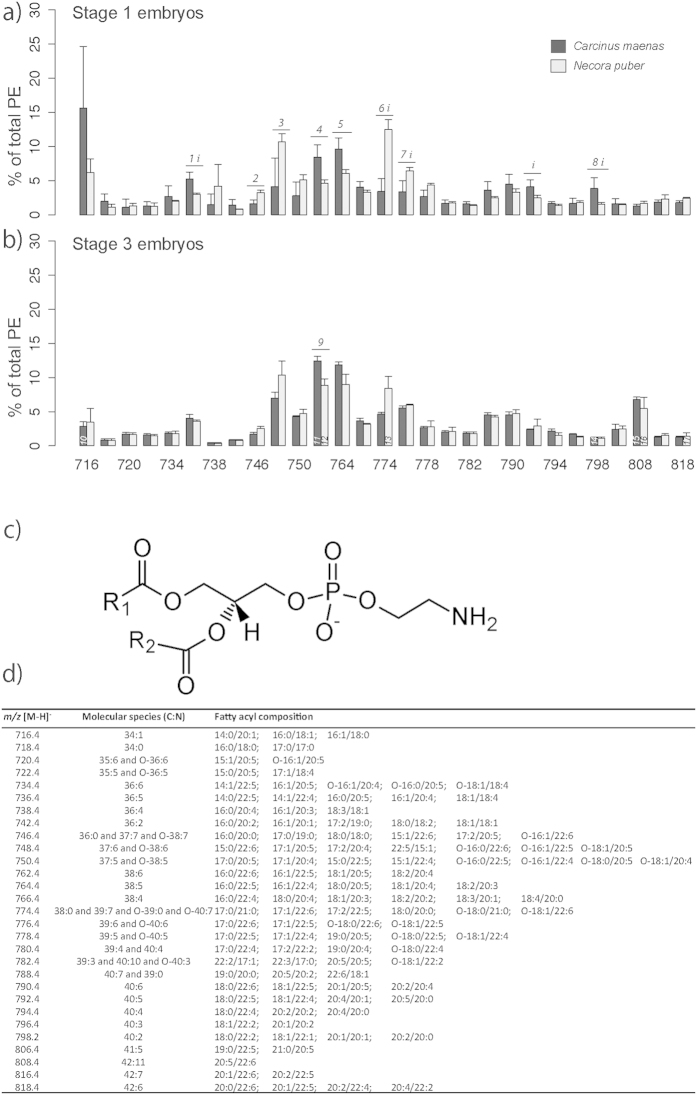
(**a**) Relative abundance of the [M-H]^−^ ions of the different molecular species of phosphatidylethanolamine (PE) present in embryos of *Carcinus maenas* and *Necora puber* at stage 1 and (**b**) at stage 3. (**c**) General structure of PE. (**d**) Major molecular species of PE identified by liquid chromatography – mass spectrometry in negative-ion mode in the embryos of *C. maenas* and *N. puber*. Error bars represent standard deviation of three independent samples. *P* values for each significant statistical test performed are represented in the figure with a number, with significant differences between crab species being represented on the top of the graph bars and significant differences between stages of the same crab species being represented within the bar of stage 3 embryos. Significant interaction between species and stage is represented with an *i* on the top of stage 1 bars. (Post hoc Tukey HSD, *1*: *P *= 0.0061; *2*: *P *= 0.0045; *3*: *P *= 0.0441; *4*: *P *= 0.0133; *5*: *P *= 0.0237; *6*: *P *= 0.0003; *7*: *P *= 0.0109; *8*: *P *= 0.0346; *9*: *P *= 0.0181; *10*: *P *= 0.0440; *11*: *P *= 0.0099; *12*: *P *= 0.0074; *13*: *P *= 0.0389; 14: *P *= 0.0167; *15*: *P *= 0.0003; *16*: *P *= 0.0032; *17*: *P *= 0.0089). Interaction species *vs* stage (*i*): *m/z* 736.4 (*P *= 0.0274); *m/z* 774.4 (*P *= 0.0146); *m/z* 776.4 (*P *= 0.0310); *m/z* 792.4 (*P *= 0.0389); *m/z* 798.2 (*P *= 0.0466).

PEs (Fig. 4c) were the most specious PL class recorded. A total of 29 molecular species were identified (Fig. 4d). In stage 1 embryos of *C. maenas* the most abundant species were at *m/z* 716.4, PE(34:1), and *m/z* 764.4, PE(38:5), (Fig. 4a), while in *N. puber* were at *m/z* 774.4, PE(38:0), PE(39:7), PE(O-39:0) or PE(O-40:7), and *m/z* 748.4, PE(37:6) or PE(O-38:6) (Fig. 4a). Stage 3 embryos of *C. maenas* displayed the ions at *m/z* 762.4, PE(38:6), and *m/z* 764.4 assigned as the most abundant molecular species, while for *N. puber* were at *m/z* 748.4 and *m/z* 764.4 (Fig. 4b). According to MS/MS analysis of the ions attributed to the PE molecular species their compositions contained saturated FA (SFA), monounsaturated FA (MUFA), polyunsaturated FA (PUFA) and highly-polyunsaturated FA (HUFA), from 14:0 to 22:6 (Fig. 4d).

**Figure 5:**
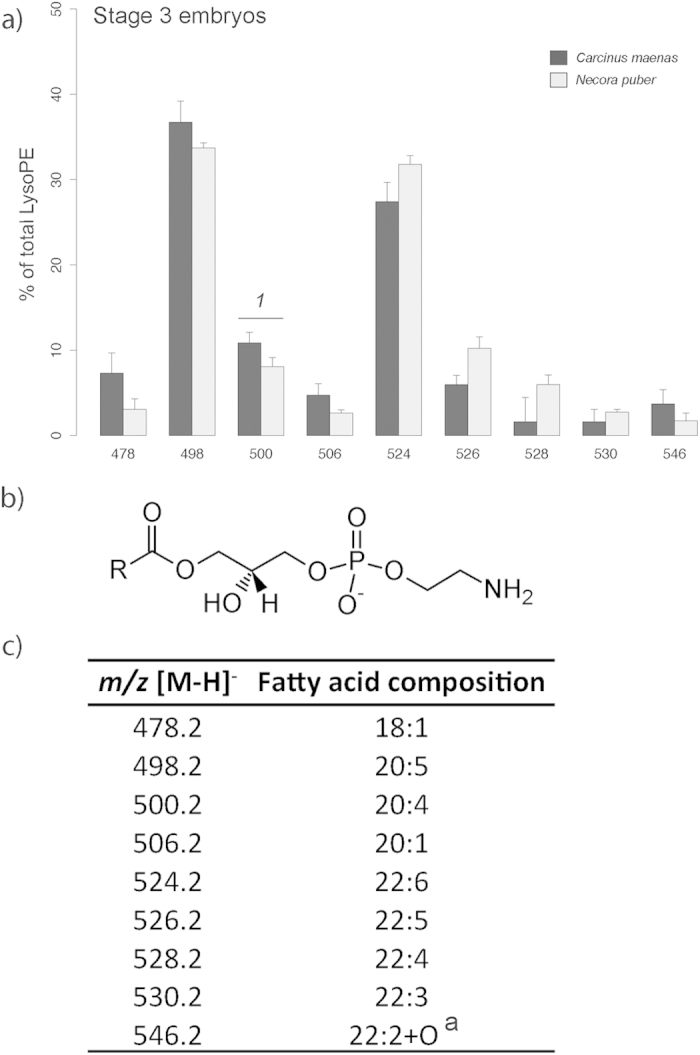
(**a**) Relative abundance of the [M-H]^−^ ions of the different molecular species of lysophosphatidylethanolamine (LysoPE) present in embryos of *Carcinus maenas* and *Necora puber* at stage 3. (**b**) General structure of LysoPE. (**c**) Major molecular species of LysoPE identified by liquid chromatography – mass spectrometry in negative-ion mode in the embryos of *C. maenas* and *N. puber*. Error bars represent standard deviation of three independent samples. *P* values for each significant statistical test performed are represented in the figure with a number, with significant differences between crab species being represented on the top of the graph bars. (*t*-test, *1*: *P *= 0.0435). ^a^12,15-epoxy-13,14-dimethyl-eicosadienoate

LysoPE (Fig. 5b) was one of the PL classes solely recorded in the last embryonic stage, with 9 molecular species (Fig. 5c) being identified in stage 3 embryos of *C. maenas* and *N. puber* (Fig. 5a). The most abundant LysoPEs recorded were at *m/z* 498.2, LysoPE(20:5), and *m/z* 524.2, LysoPE(22:6), (Fig. 5a).

### Sphingomyelin (SM)

**Figure 6:**
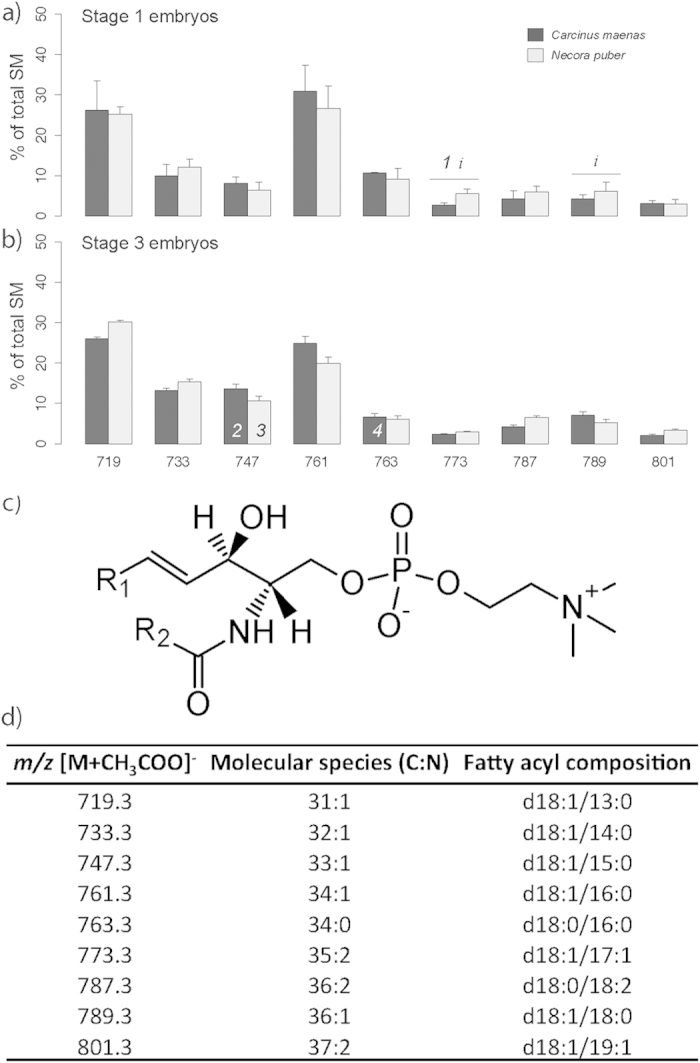
(**a**) Relative abundance of the [M+CH_3_COO]^−^ ions of the different molecular species of sphingomyelin (SM) present in embryos of *Carcinus maenas* and *Necora puber* at stage 1 and (b) at stage 3. (**c**) General structure of SM. (**d**) Major molecular species of SM identified by liquid chromatography – mass spectrometry in negative-ion mode in the embryos of *C. maenas* and *N. puber*. Error bars represent standard deviation of three independent samples. *P* values for each significant statistical test performed are represented in the figure with a number, with significant differences between crab species being represented on the top of the graph bars and significant differences between stages of the same crab species being represented within the bar of stage 3 embryos. Significant interaction between species and stage is represented with an *i* on the top of stage 1 bars. (Post hoc Tukey HSD, *1*: *P *= 0.0043; *2*: *P *= 0.0115; *3*: *P *= 0.0440; *4*: *P *= 0.03889). Interaction species *vs* stage (*i*): *m/z* 773.3 (*P *= 0.0223); *m/z* 789.3 (*P *= 0.0449).

The LC-MS/MS spectra of SMs (Fig. 6c) revealed the presence of 9 molecular species (Fig. 6d). For both *C. maenas* and *N. puber*, the most abundant molecular species at stage 1 and 3 embryos were at *m/z* 761.3, SM(d18:1/16:0, with the FAs in the positions *sn*-1 and *sn*-2, respectively) and *m/z* 719.3, SM(d18:1/13:0), (Fig. 6a,b). The SM species presented a fatty acyl combination of SFA and MUFA, with one PUFA. The FAs present in this PL class ranged from 13:0 to 19:1 (Fig. 6d).

### Phosphatidylinositol (PI)

**Figure 7:**
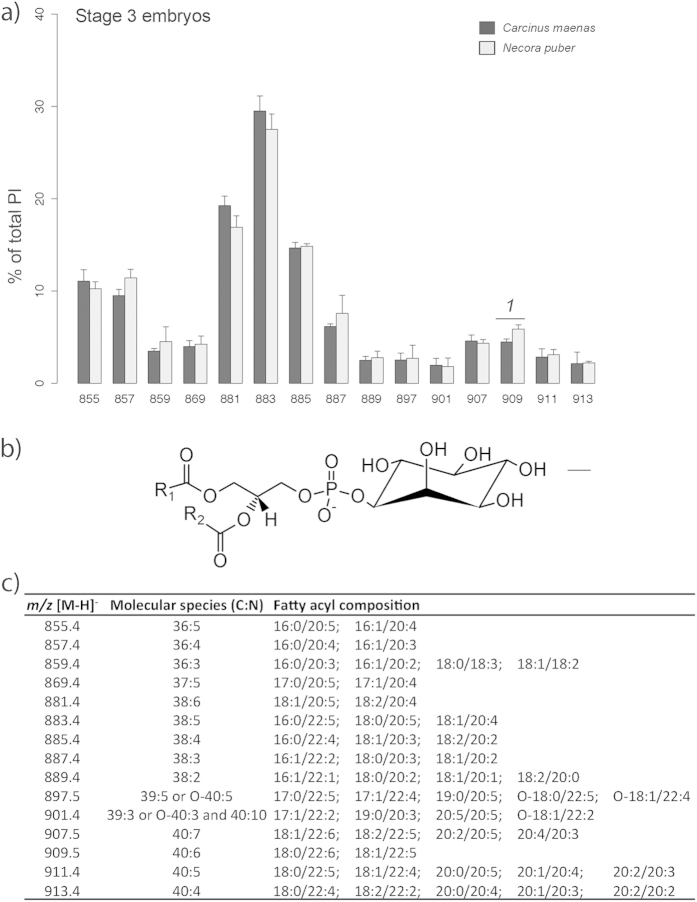
(**a**) Relative abundance of the [M-H]^−^ ions of the different molecular species of phosphatidylinositol (PI) present in embryos of *Carcinus maenas* and *Necora puber* at stage 3. (**b**) General structure of PI. (**c**) Major molecular species of PI identified by liquid chromatography – mass spectrometry in negative-ion mode in the embryos of *C. maenas* and *N. puber*. Error bars represent standard deviation of three independent samples. *P* values for each significant statistical test performed are represented in the figure with a number, with significant differences between crab species being represented on the top of the graph bars. (*t*-test, *1*: *P *= 0.0175).

PIs (Fig. 7b) were one of the PL classes that were synthesized during embryogenesis, thus solely being recorded in stage 3 embryos. The LC-MS analysis identified a total of 15 molecular species (Fig. 7c), with the ions at *m/z* 883.4, PI(38:5), and *m/z* 881.4, PI(38:6), being the most abundant in both crab species (Fig. 7a). The fatty acyl composition of PIs included SFA, MUFA, PUFA and HUFA ranging from 16:0 to 22:6 (Fig. 7c).

### Cardiolipin (CL)

**Figure 8:**
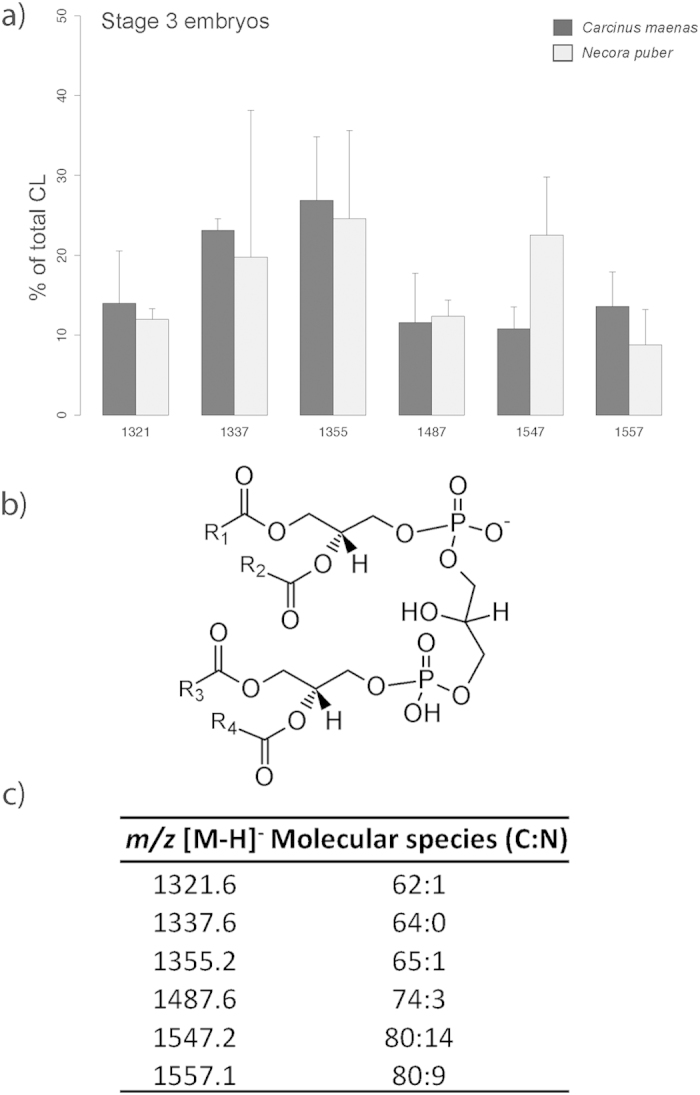
(**a**) Relative abundance of the [M-H]^−^ ions of the different molecular species of cardiolipin (CL) present in embryos of *Carcinus maenas* and *Necora puber* at stage 3. (**b**) General structure of CL. (**c**) Major molecular species of CL identified by liquid chromatography – mass spectrometry in negative-ion mode in the embryos of *C. maenas* and *N. puber*. Error bars represent standard deviation of three independent samples.

CLs (Fig. 8b) only appeared in the last embryonic stage. A total of 6 molecular species were identified (Fig. 8c), the most abundant being at *m/z* 1355.2, CL(65:1), and *m/z* 1337.6, CL(64:0), in *C. maenas* and at *m/z* 1355.2 and *m/z* 1547.2, CL(80:14) in *N. puber* (Fig. 8a).

### Analysis of the FA profile

The identification of the FA profile of the total lipid extract was performed by GC-MS analysis of FA methyl esters (FAMEs). This analysis corroborated the information on the FAs recorded in the PL classes described above. The relative quantification of FAs revealed that palmitic acid (16:0) was the most abundant FA in both embryonic stages of the two sympatric crab species (see supplementary Table S5 for a complete list of all FAs recorded). In *C. maenas*, the major FAs were 16:0 (16.31 ± 0.39%), 16:1*n*7 (15.37 ± 3.24%) at stage 1 and 16:0 (18.60 ± 1.28%), eicosapentaenoic acid (EPA, 20:5*n*-3) (14.32 ± 0.75%) at stage 3. However, in *N. puber* the most abundant FAs were 16:0 (18.86 ± 0.33%, 19.57 ± 0.34%) and 16:1*n*7 (17.21 ± 1.14%, 12.82 ± 2.91%) at stage 1 and 3 embryos, respectively. The analysis of FA classes (Fig. 1c,d) showed that MUFA were the most well represented in the pool of FAs (*C. maenas*: 38.51 ± 4.45%, 33.98 ± 1.05%; *N. puber* 38.26 ± 0.46%, 34.71 ± 3.47%, at stage 1 and 3, respectively), followed by HUFA (*C. maenas*: 23.95 ± 3.96%, 28.23 ± 2.76%; *N. puber* 20.61 ± 1.40%, 23.73 ± 2.16%, at stage 1 and 3, respectively) and SFA (*C. maenas*: 23.45 ± 1.75,% 27.34 ± 1.27%; *N. puber* 26.33 ± 0.68%, 21.61 ± 1.29%, at stage 1 and 3, respectively). Within the HUFA, EPA (*C. maenas*: 9.02 ± 2.06%, 14.32 ± 0.75%; *N. puber* 8.17 ± 0.59%, 10.11 ± 1.10%, at stage 1 and 3, respectively) and docosahexaenoic acid (DHA, 22:6*n*-3) (*C. maenas*: 9.36 ± 4.02%, 9.63 ± 2.02%; *N. puber* 7.58 ± 0.83%, 8.01 ± 1.48%, stage 1 and 3, respectively) were the most abundant FAs.

## Discussion

The lipidomic approach employed in the present study allowed an unprecedented insight on the lipid dynamics during embryonic development in decapod crustaceans. TLC analysis revealed the biochemical evolution of lipids during embryogenesis at cellular level. PL classes present at stage 1 (PC, LysoPC, PE, SM) commonly play a key role on energetic and structural functions, while those that solely were recorded at stage 3 (LysoPE, PI and CL) may be involved in the neurologic and sensorial development of embryos about to hatch^13^.

The important role that PC plays during embryogenesis was reflected in our results, with their decrease in relative abundance from stage 1 to 3 embryos agreeing with data from other marine species^14^,^15^. PC is recognized as the major component of biological membranes^16^, therefore of paramount importance on cells biochemistry and physiology^17^,^18^. Additionally, PC also has a similar role in the transport of yolk components in developing embryos^15^. Although the catabolism of PC may provide a secondary energy source when TAG are depleted^4^, it is most likely related with the provision of phosphate and choline. PCs likely play an important function as a source of EFAs for developing embryos^15^. PCs are rich in EPA and DHA, which are both required for cell differentiation and membrane formation during embryogenesis^19^. In line with the above, a possible consequence of excessive or even complete catabolism of PLs for energy would be the loss of important HUFA. However, studies carried out on fish embryos and larvae showed a selective retention of the DHA liberated by the catabolism of PCs in neutral lipids and/or PEs^15^,^20^. Such EFAs are also known to play a major role during late embryonic development and early larval life in brachyuran crabs^21^. The low variability in the relative abundance of PEs between embryonic stages, in both crab species, suggests a structural function of this PL class. PC and PE were the major components of polar lipids. As already referred above for PC, PE is also a key component of membrane bilayers. Moreover, the FA composition of the molecular species of PEs showed a high level of unsaturation, which is known to contribute to membrane fluidity, as this feature is largely determined by the degree of FA unsaturation and *n*-3 HUFA^22^. Specifically, the role played by DHA and EPA on membrane flexibility has already been documented^22^. In both *C. maenas* and *N. puber*, the molecular species of this class that display significant differences between stage 1 and 3 embryos (*m/z* 762.4 and *m/z* 808.4) possess EPA, DHA and/or arachidonic acid (ARA, 20:4*n*-6) in their composition. The selective retention of these FAs in the last stages of embryonic development may reflect a preparation for planktonic life. In decapod crustaceans, those EFAs are known to be decisive for larval fitness during early planktonic life stages^3^. These molecules influence early neural development and functions^23^, hatchability at the end of embryonic development^24^, larval growth^25^ and larval resistance to stress^26^. Other molecular species of LysoPEs include C_20_ and C_22_ HUFA, important FAs, such as ARA, EPA, DHA, docopentaenoic acid (DPA, 22:5) and 12,15-epoxy-13,14-dimethyl-eicosadienoate (Fig. 5c), which are known to be precursors of key biomolecules. ARA and EPA are the precursors of highly bioactive derivatives, the eicosanoids^7^, whereas ARA is the precursor of prostaglandins, which are known to be involved in a number of vital pathways (*e.g*., reproduction, digestion, respiration, membrane permeability and fat dissolution)^27^.

PIs and CLs were not recorded in early stage embryos and thus must originate from the catabolism of yolk reserves provided through maternal investment. PIs are considered as key signalling molecules and are involved in secretory events, as well as intercellular signalling events^28^. The metabolism of inositol lipids is involved in the signal transduction of many hormones, neurotransmitters and growth factors^29^,^30^. Qualitative requirements suggest that PC may be more important for growth, while PI may be relevant for organogenesis and tissue differentiation^7^. These features may explain their occurrences in different periods of embryogenesis (*e.g*., PCs are already present in newly extruded embryos).

In general, the analysis of the lipidomic profiles of developing embryos of *C. maenas* and *N. puber* did not reveal the existence of major interspecific differences. In the sampling site, these two species share the same estuarine habitats, which allow us to infer that they may have access to the same dietary resources. The scarce differences in the lipidomic profiles of embryos in the beginning of their development (stage 1) demonstrate a similar maternal investment in both crab species. Embryos of *C. maenas* and *N. puber* also appear to be programed to catabolize embryonic resources in a similar pattern during the incubation period, as the incubation environment (in terms of female brooding chamber) is similar for both species^31^,^32^. A follow up study surveying ovigerous females from these two species where they no longer occur in sympatry (*C. maenas* present in the inner regions of the coastal lagoon, which are more strongly influenced by fresh water runoffs, and *N. puber* located in a typically marine environment, the outer regions of the pier protecting the inlet of the coastal lagoon) will allow us to determine if the trends recorded in maternal investment and lipid catabolism during embryogenesis remain similar under contrasting environmental conditions.

Furthermore, the analysis of FA classes revealed scarce differences between both crab species. Decapod larvae generally exhibit a limited ability to introduce double bonds into the *n*-6 and *n*-3 position of C_18_, C_20_, and C_22_ FAs^33^. The presence of EPA and DHA in newly hatched larvae is known to originate from maternal lipids transferred at oogenesis, as decapod embryos are lecitothrophic and cannot synthesize these FAs *de novo*^34^,^35^. The selective retention of essential HUFA through embryogenesis may thus reduce the nutritional vulnerability of newly hatched larvae to suboptimal conditions they may experience in the early stages of their planktonic life^3^. High levels of HUFA and PL improve the osmoregulation process by optimizing membranous lipid composition and/or structure of the gills^8^, a key feature for larvae as the ones of *C. maenas* and *N. puber* that may hatch in estuarine systems prone to salinity shifts. Since HUFA are essential components in the nutrition of decapod larvae, the lipid composition of last embryonic stages may serve as an indicator of the physiological condition of larvae about to hatch. Additionally, lipid content in embryos can potentially determine early larval success and optimal development^36^. Higher lipid content in pre-hatching embryos is commonly interpreted as sign of superior tolerance by larvae to longer periods of starvation before first feeding^37^. It must be highlighted that to date no study has ever tried to link the performance of newly hatched larvae to the lipidome displayed by developing embryos. Therefore, until these studies are made available, any assumption on the superior/inferior quality of newly hatched larvae solely based on embryos lipidomics remains speculative and probably misleading. The unprecedented level of resolution achieved on polar lipids dynamics using lipidomics opens a new research window for studying maternal investment and resource partitioning in marine organisms.

## Methods

### Sampling

Ovigerous females of *C. maenas* and *N. puber* (carapace width, average ± SD, 50.2 ± 2.5 mm) were collected in the mussel beds of Mira Channel, Ria de Aveiro (Portugal) (40°38′26.30“N, 8°43′58.90“W) during March (early spring) 2012. Embryos were classified according to Rosa *et al*.^5^: stage 1 (newly extruded embryos) - uniform yolk, no cleavage or eyes; stage 3 (embryos ready to hatch in <48 h) nearly no yolk present and embryo fully developed. Three females carrying embryos at stage 1 and three carrying embryos at stage 3 were selected for each species and their egg mass removed with fine forceps. All collected samples were freeze-dried and stored at –32 °C for biochemical analysis.

### Lipid extraction

The Bligh and Dyer method^38^ was used to isolate total lipids from embryos. Samples were resuspended in glass centrifuge tubes using 1 mL of water and 3.75 mL of chloroform/methanol (1:2, V/V) and incubated on ice for 30 min. The samples were centrifuged at 2000 rpm for 10 min at room temperature to resolve a two–phase system: an aqueous upper phase which contained the non-lipid components and an organic lower phase from where the lipids were recovered. The extraction was repeated twice. The organic phases were dried under a nitrogen stream. Lipid extracts were preserved at −20 °C for further analysis.

### Quantification of PL by phosphorus assay

Quantification of PL in the total lipid extract and in the spots separated by TLC was performed according to Bartlett and Lewis^39^. Samples were put on acid-washed glass tubes and resuspended in 0.650 mL of perchloric acid (70%, m/V). Glass tubes were incubated for 60 min at 180 °C in a heating block. After incubation, 3.3 mL of water, 0.5 mL of ammonium molybdate (2.5%, m/V) and 0.5 mL of ascorbic acid (10%, m/v) were added. After each addition, the mixture was well homogenized in a vortex mixer and incubated during 5 min at 100 °C in a water bath. Standards from 0.1 to 3.0 μg of phosphate (standard solution of NaH_2_PO_4_. 2H_2_O, 439 mg L^−1^ of water, *i.e*. 100 μg of phosphorus mL^−1^) underwent the same treatment as the samples. Absorbance of standards and samples was measured at 800 nm, at room temperature, in a microplate UV-vis spectrophotometer. The relative abundance of each PL class was calculated by the relation of the amount of phosphorus in each spot to the phosphorus amount of the total lipid extract in the sample applied in the TLC spot.

### Separation of PL classes by TLC

The TLC method was used to separate the PL from the total lipid extract using TLC silica gel plates with concentration zone. Initially, the plates were washed with chloroform/methanol (1:1, V/V) and activated (sprayed) with 2.3% of boric acid in ethanol and dried for 30 min at 100 °C in an oven. The samples (20 μL of chloroform solution containing 30 μg of PL) were applied on the TLC plate and eluted with chloroform/ethanol/water/triethylamine (30:35:7:35, V/V/V/V). After the elution, the PL spots were revealed by spraying with a primuline solution (50 μg in 10 mL of acetone/water, 80:20, V/V) and visualized with a UV lamp (246 and 366 nm)^40^,^41^. The identification of PL spots was accomplished by using PL standards (PC, PE, SM, LysoPC, PI, CL) from Avanti® Polar Lipids, Inc. (Alabaster, AL, USA), applied on the TLC plate. Spots coincident with the migration of standards were scraped into glass tubes and quantified as described above.

### HILIC-ESI-MS of the total lipid extracts

HILIC analysis of total lipid extracts was performed on a Waters Alliance 2690 HPLC system (Waters Corp., Milford, MA, USA) coupled to a Finnigan LXQ electrospray linear ion trap mass spectrometer (Thermo Fisher, San Jose, CA, USA). Mobile phase A consisted of 50% acetonitrile, 25% methanol, and 25% water with 10 mM ammonium acetate, and mobile phase B consisted of 60% acetonitrile and 40% methanol with 10 mM ammonium acetate. The lipid extracts (25 μg) were diluted in mobile phase B (90 μL) and 10 μL of the reaction mixture was introduced into an Ascentis Si HPLC Pore column (150 mm × 1.0 mm, 3 μm; Sigma-Aldrich). The solvent gradient was programmed as follows: gradient started with 0% of A and 100% of B, linearly increased to 100% of A in 20 min, and isocratically held for 35 min, returning to the initial conditions in 5 min. The flow rate through the column was 7.5 μL min^−1^ obtained using a pre-column split (Accurate, LC Packings, San Francisco, CA, USA). PL internal standards were purchased from Avanti® Polar Lipids, Inc. (Alabaster, AL, USA) and used without further purification: 1′,3′-bis[1,2-dimyristoyl-*sn*-glycero-3-phospho]-*sn*-glycerol (CL); 1,2-dimyristoyl-*sn*-glycero-3-phosphocholine (dMPC); 1-nonadecanoyl-2-hydroxy-*sn*-glycero-3-phosphocholine (LysoPC); 1,2-dimyristoyl-*sn*-glycero-3-phosphoethanolamine (dMPE); 1,2-dipalmitoyl-*sn*-glycero-3-phospho-(1′-myo-inositol) (dPPI); and N-(heptadecanoyl)-sphing-4-enine-1-phosphocholine (SM). Polar lipid analysis was carried out by negative-ion electrospray ionization mass spectrometry (ESI-MS) on the Finnigan LXQ linear ion trap mass spectrometer. The electrospray voltage was 4.7 kV, the capillary temperature was 275 °C, and the sheath gas (He) flow rate was 25 units. A precursor ion isolation width of 0.5 *m/z* units was used, with a 30 ms activation time for MS/MS experiments. Full scan MS spectra and MS/MS spectra were acquired with a maximum ionization time of 50 ms and 200 ms, respectively. The normalized collision energy (CE) varied between 17 and 20 (arbitrary units) for MS/MS. The data were acquired and the results were treated with the Xcalibur^®^ Data System 2.0 (Thermo Scientific, San Jose, CA, USA)^42^.

### FA analysis by GC-MS

Total FAs were analyzed by GC-MS after transesterification of embryos’ total lipid extracts (20 μg of total PL). FAMEs were prepared using a methanolic solution of potassium hydroxide (2.0 M) according to the previously described method^43^. FAMEs were resuspended in 40 μL of hexane, with 2 μL of this hexane solution being used for GC-MS analysis on an Agilent Technologies 6890N Network (Santa Clara, CA) equipped with a DB-1 column with 30 m of length, 0.25 mm of internal diameter, and 0.1 μm of film thickness (J&W Scientific, Folsom, CA). The GC was connected to an Agilent 5973 Network Mass Selective Detector operating with an electron impact mode at 70 eV and scanning the range *m/z* 40–500 in a 1s cycle in a full scan mode acquisition. The oven temperature was programmed from an initial temperature of 90 °C, standing at this temperature for 0.5 min and following a linear increase to 220 °C at 20 °C/min, a linear increase at 2 °C/min to 240 °C, and 5 °C/min until reaching 250 °C. The injector and detector temperatures were 220 and 230 °C, respectively. Helium was used as the carrier gas at a flow rate of 1.7 mL/min. FAME identification was performed by comparing their retention time and mass spectrum, which was analysed with MS spectra of commercial FAME standards (Supelco 37 Component FAME Mix) and confirmed by comparison with the chemical database Wiley and the spectral library “The AOCS Lipid Library”^44^. FA profile was classified in six FA classes: SFA, MUFA, PUFA, HUFA, Branched FA (BrFA)), Cyclic FA (CyFA) and Epoxy FA (EpFA). While polyunsaturated FAs are commonly defined as all FAs with ≥2 double bonds, in the present study we distinguish between PUFA (FAs with 2 or 3 double bonds) and HUFA (FAs with ≥4 double bonds).

### Statistical analysis

For PL quantification by LC-MS, the area of each molecular species in the spectra was transformed in their relative abundance, using the area of the PL internal standards as reference. The relative content of each PL class and FA was calculated as percentage of total PL and FA profiles, respectively. Differences in the relative abundance of PL classes, FA classes and molecular species of PL, were determined by 2-way ANOVA with interaction (factor crab species: *C. maenas* and *N. puber*; factor stage: stage1 and stage 3). Post hoc Tukey HSD test was used when ANOVA results revealed significant differences (*P *< 0.05). Statistical analyses of PL classes and their molecular species present in only one embryonic stage were performed using pairwise comparisons (Student´s *t*-test) between crab species. The sample size was identical in all treatments: n* *= 3. Prior to analysis, we tested for deviations from normality in the response variable with the Shapiro test and homogeneity of variance with the Levene’s test. The level of statistical significance was *P *< 0.05. The statistical analyses were performed using the statistical package R version 2.13.2^45^.

## Additional Information

**How to cite this article**: Rey, F. *et al*. Unravelling polar lipids dynamics during embryonic development of two sympatric brachyuran crabs (*Carcinus maenas* and *Necora puber*) using lipidomics. *Sci. Rep*. **5**, 14549; doi: 10.1038/srep14549 (2015).

## Supplementary Material

Supplementary Information
