# Supplementary material for: Unravelling polar lipids dynamics during embryonic development of two sympatric brachyuran crabs (Carcinus maenas and Necora puber) using lipidomics
Source: Sci Rep. 2015 Sep 30;5:14549. doi: 10.1038/srep14549 (PMC4588508; doi:10.1038/srep14549)
Supplement: Supplementary Information [file srep14549-s1.pdf]

Unravelling polar lipids dynamics during embryonic development of two sympatric brachyuran crabs (*Carcinus maenas* and *Necora puber*) using lipidomics

Felisa Rey[1]*, Eliana Alves[2], Tânia Melo[2], Pedro Domingues[2], Henrique Queiroga[1], Rui Rosa[3], M. Rosário M. Domingues[2], Ricardo Calado[1]*

[1] Departamento de Biologia & CESAM, Universidade de Aveiro, Campus Universitário de Santiago, 3810-193 Aveiro, Portugal

[2] Mass Spectrometry Centre, Department of Chemistry & QOPNA, Universidade de Aveiro, Campus Universitario de Santiago, 3810-193 Aveiro, Portugal

[3] MARE – Marine and Environmental Sciences Centre, Laboratório Marítimo da Guia, Faculdade de Ciências da Universidade de Lisboa, Av. Nossa Senhora do Cabo, 939, 2750-374 Cascais, Portugal

Corresponding Authors:

*Ricardo Calado

Phone + 351 234 370 779

E-mail: rjcalado@hotmail.com

*Felisa Rey

E-mail: felisa.rey@gmail.com

Supplementary Figure S1: (S1.1) LC-MS chromatograms of *Carcinus maenas* embryos at stage 1 and (S1.2) stage 3, (S1.3) LC-MS chromatograms of *Necora puber* embryos at stage 1 and (S1.4) stage 3.

Carcinus maenas

S1.1

Stage 1

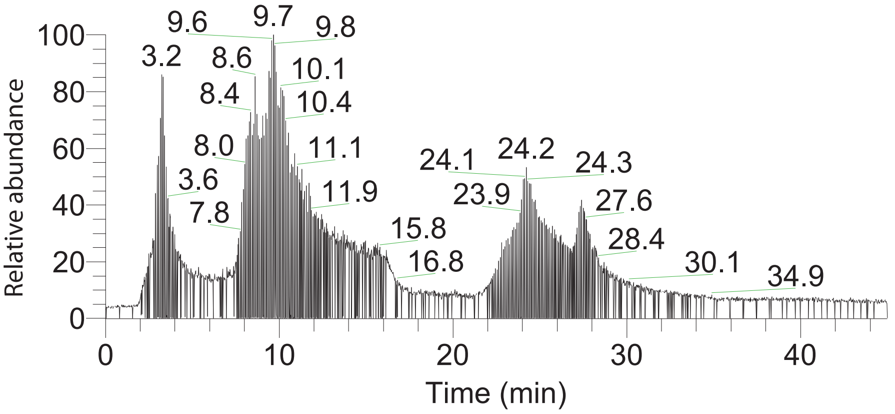

S1.2

Stage 3

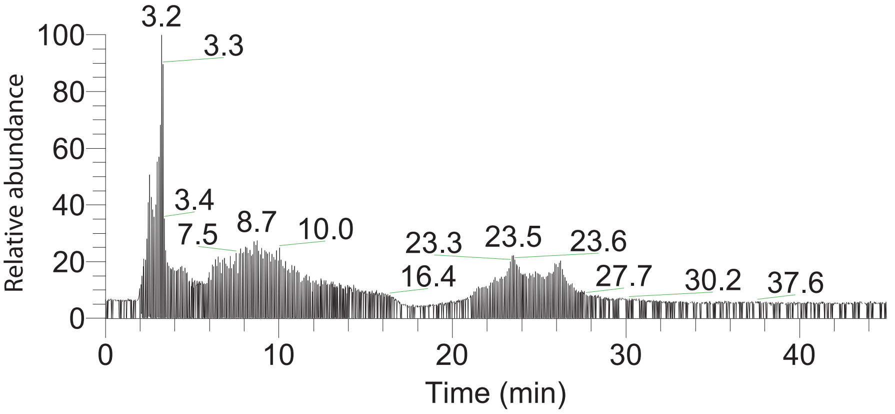

Necora puber

S1.3

Stage 1

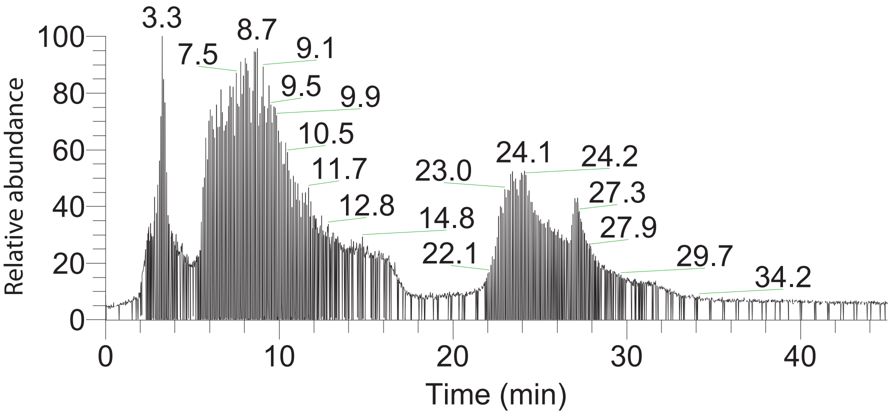

S1.4

Stage 3

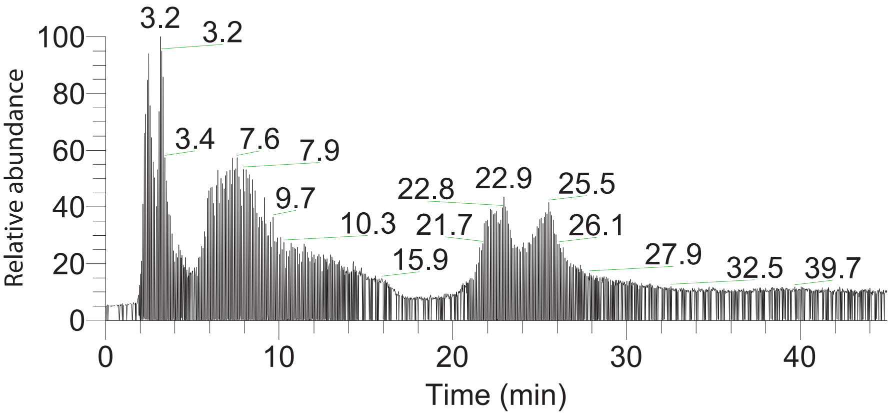

Supplementary Figure S2: MS/MS spectra of *Carcinus maenas* embryos at stage 1 and stage 3. Abbreviations: PC – phosphatidylcholine; LysoPC – lysophosphatidylcholine; PE – phosphatidylethanolamine; LysoPE – lysophosphatidylethanolamine; SM – sphingomyelin; PI – phosphatidylinositol; CL – cardiolipin.

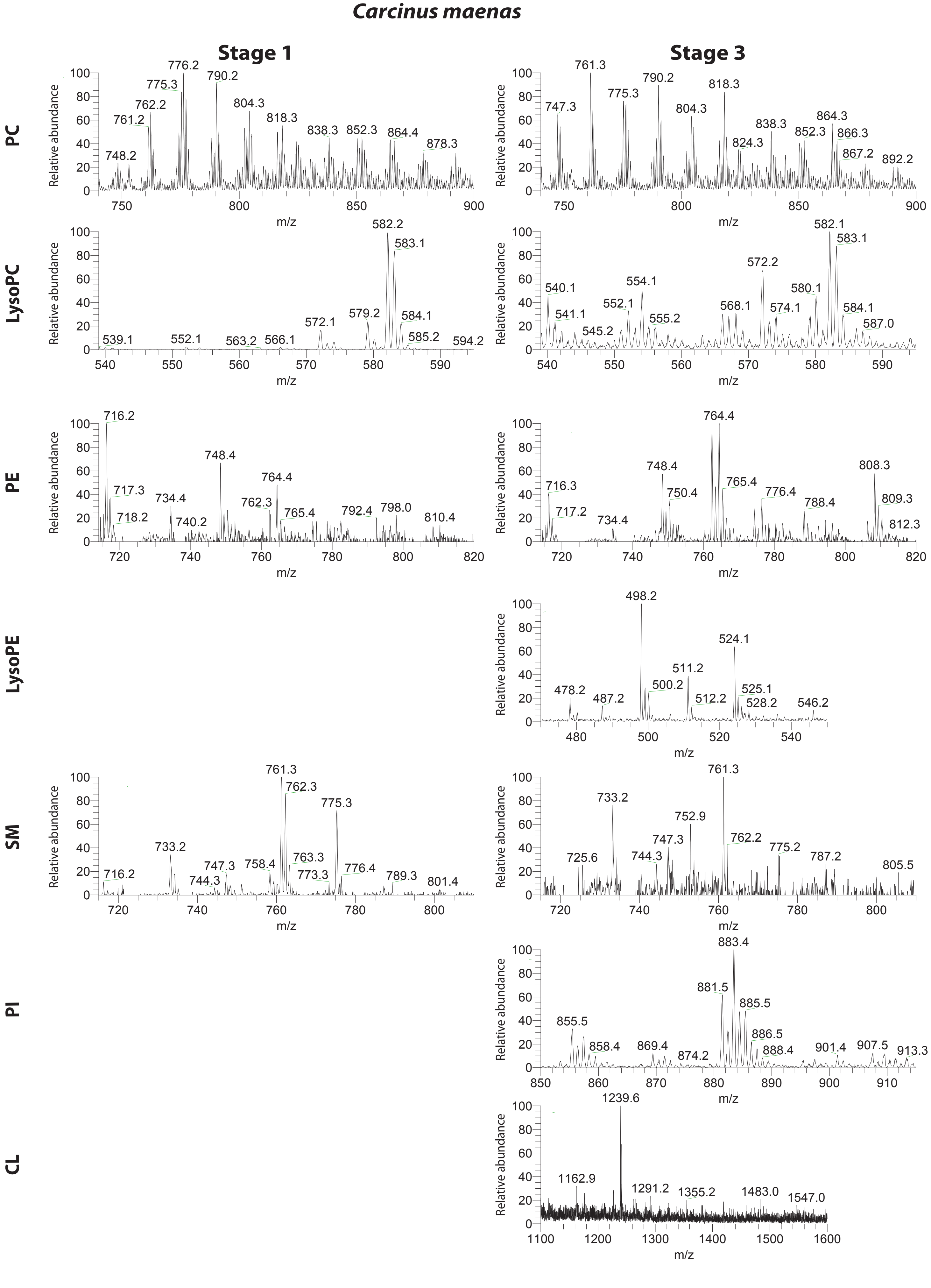

Supplementary Figure S3: MS/MS spectra of *Necora puber* embryos at stage 1 and stage 3. Abbreviations: PC – phosphatidylcholine; LysoPC – lysophosphatidylcholine; PE – phosphatidylethanolamine; LysoPE – lysophosphatidylethanolamine; SM – sphingomyelin; PI – phosphatidylinositol; CL – cardiolipin.

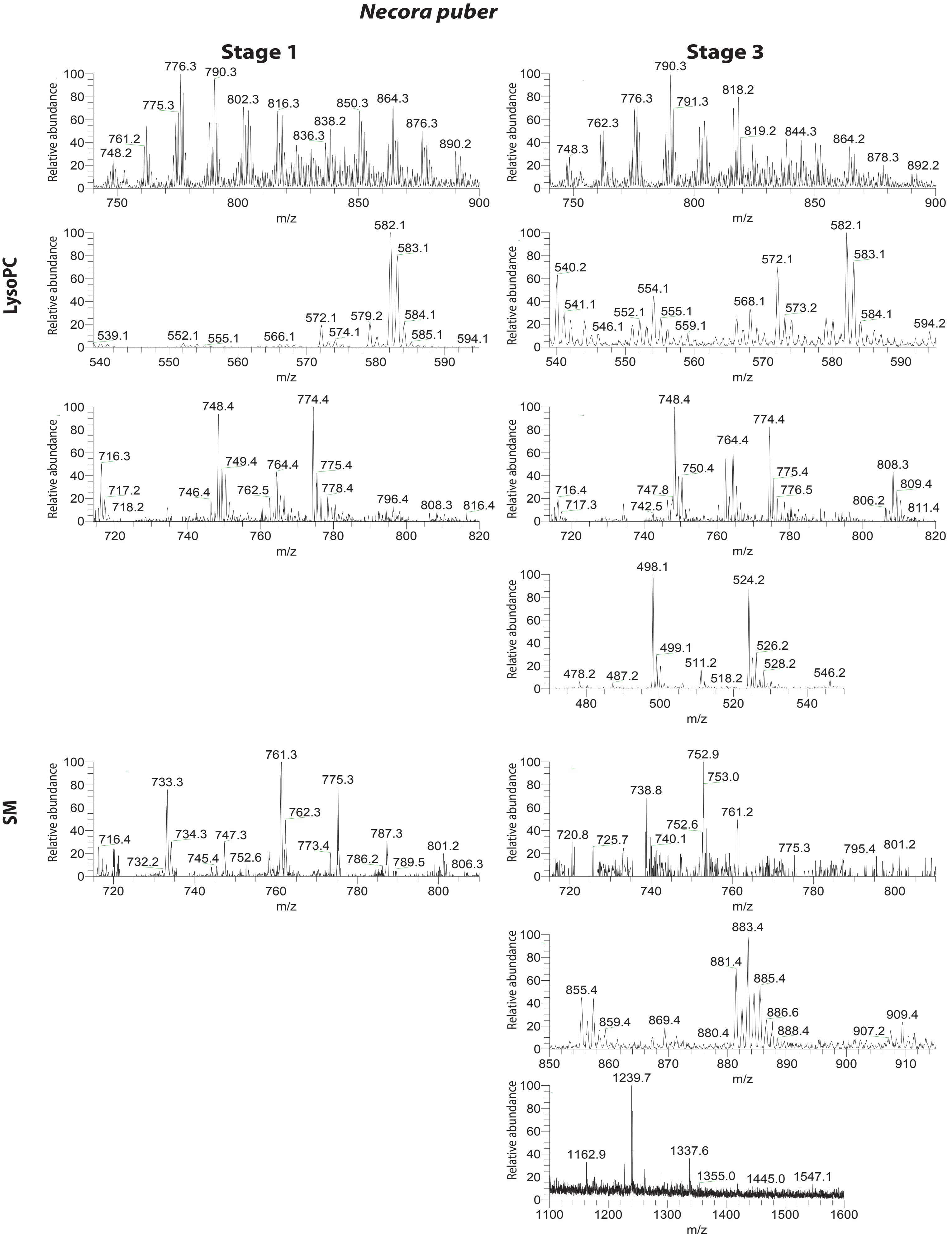

Supplementary Table S4: Total molecular species identified by LC-MS in negative-ion mode.

m/z	m/z	Phospholipid Class	Molecular species (C:N)	Fatty acyl composition					
478.2	[M-H]$^{-}$	LysoPE	18:1						
498.2	[M-H]$^{-}$	LysoPE	20:5						
500.2	[M-H]$^{-}$	LysoPE	20:4						
506.2	[M-H]$^{-}$	LysoPE	20:1						
524.2	[M-H]$^{-}$	LysoPE	22:6						
526.2	[M-H]$^{-}$	LysoPE	22:5						
528.2	[M-H]$^{-}$	LysoPE	22:4						
530.2	[M-H]$^{-}$	LysoPE	22:3						
540.1	[M+CH3COO]$^{-}$	LysoPC	15:0 or O-16:0						
546.2	[M-H]$^{-}$	LysoPE	22:2+O-*						
552.1	[M+CH3COO]$^{-}$	LysoPC	16:1						
554.1	[M+CH3COO]$^{-}$	LysoPC	16:0						
566.1	[M+CH3COO]$^{-}$	LysoPC	17:1 or O-18:1						
574.1	[M+CH3COO]$^{-}$	LysoPC	18:4						
580.1	[M+CH3COO]$^{-}$	LysoPC	18:1						
582.1	[M+CH3COO]$^{-}$	LysoPC	18:0						
594.1	[M+CH3COO]$^{-}$	LysoPC	19:1						
716.4	[M-H]$^{-}$	PE	34:1	14:0/20:1;	16:0/18:1;	16:1/18:0			
718.4	[M-H]$^{-}$	PE	34:0	16:0/18:0;	17:0/17:0				
719.3	[M+CH3COO]$^{-}$	SM	31:1	d18:1/13:0					
720.4	[M-H]$^{-}$	PE	35:6 and O-36:6	15:1/20:5;	O-16:1/20:5				
722.4	[M-H]$^{-}$	PE	35:5 and O-36:5	15:0/20:5;	17:1/18:4				
733.3	[M+CH3COO]$^{-}$	SM	32:1	d18:1/14:0					
734.4	[M-H]$^{-}$	PE	36:6	14:1/22:5;	16:1/20:5;	O-16:1/20:4;	O-16:0/20:5;	O-18:1/18:4	
736.4	[M-H]$^{-}$	PE	36:5	14:0/22:5;	14:1/22:4;	16:0/20:5;	16:1/20:4;	18:1/18:4	
738.4	[M-H]$^{-}$	PE	36:4	16:0/20:4;	16:1/20:3;	18:3/18:1			
742.4	[M-H]$^{-}$	PE	36:2	16:0/20:2;	16:1/20:1;	17:2/19:0;	18:0/18:2;	18:1/18:1	
746.4	[M-H]$^{-}$	PE	36:0 and 37:7 and O-38:7	16:0/20:0;	17:0/19:0;	18:0/18:0;	15:1/22:6;	17:2/20:5;	O-16:1/22:6
747.3	[M+CH3COO]$^{-}$	SM	33:1	d18:1/15:0					
748.2	[M+CH3COO]$^{-}$	PC	29:1 or O-30:1						

m/z	m/z	Phospholipid Class	Molecular species (C:N)	Fatty acyl composition							
748.4	[M-H]$^{-}$	PE	37:6 and O-38:6	15:0/22:6;	17:1/20:5;	17:2/20:4;	22:5/15:1;	O-16:0/22:6;	O-16:1/22:5	O-18:1/20:5	
750.4	[M-H]$^{-}$	PE	37:5 and O-38:5	17:0/20:5;	17:1/20:4;	15:0/22:5;	15:1/22:4;	O-16:0/22:5;	O-16:1/22:4	O-18:0/20:5	O-18:1/20:4
761.3	[M+CH3COO]$^{-}$	SM	34:1	d18:1/16:0							
762.3	[M+CH3COO]$^{-}$	PC	30:1 or O-31:1								
762.4	[M-H]$^{-}$	PE	38:6	16:0/22:6;	16:1/22:5;	18:1/20:5;	18:2/20:4				
763.3	[M+CH3COO]$^{-}$	SM	34:0	d18:0/16:0							
764.4	[M-H]$^{-}$	PE	38:5	16:0/22:5;	16:1/22:4;	18:0/20:5;	18:1/20:4;	18:2/20:3			
766.4	[M-H]$^{-}$	PE	38:4	16:0/22:4;	18:0/20:4;	18:1/20:3;	18:2/20:2;	18:3/20:1;	18:4/20:0		
773.3	[M+CH3COO]$^{-}$	SM	35:2	d18:1/17:1							
774.4	[M-H]$^{-}$	PE	38:0 and 39:7 and O-39:0 and O-40:7	17:0/21:0;	17:1/22:6;	17:2/22:5;	18:0/20:0;	O-18:0/21:0;	O-18:1/22:6		
776.3	[M+CH3COO]$^{-}$	PC	31:1 or O-32:1								
776.4	[M-H]$^{-}$	PE	39:6 and O-40:6	17:0/22:6;	17:1/22:5;	O-18:0/22:6;	O-18:1/22:5				
778.3	[M+CH3COO]$^{-}$	PC	31:0 or O-32:0								
778.4	[M-H]$^{-}$	PE	39:5 and O-40:5	17:0/22:5;	17:1/22:4;	19:0/20:5;	O-18:0/22:5;	O-18:1/22:4			
780.4	[M-H]$^{-}$	PE	39:4 and 40:4	17:0/22:4;	17:2/22:2;	19:0/20:4;	O-18:0/22:4				
782.4	[M-H]$^{-}$	PE	39:3 and 40:10 and O-40:3	22:2/17:1;	22:3/17:0;	20:5/20:5;	O-18:1/22:2				
787.3	[M+CH3COO]$^{-}$	SM	36:2	d18:0/18:2							
788.3	[M+CH3COO]$^{-}$	PC	32:2 or O-33:2								
788.4	[M-H]$^{-}$	PE	40:7 and 39:0	19:0/20:0;	20:5/20:2;	22:6/18:1					
789.3	[M+CH3COO]$^{-}$	SM	36:1	d18:1/18:0							
790.3	[M+CH3COO]$^{-}$	PC	32:1 or O-33:1								
790.4	[M-H]$^{-}$	PE	40:6	18:0/22:6;	18:1/22:5;	20:1/20:5;	20:2/20:4				
792.4	[M-H]$^{-}$	PE	40:5	18:0/22:5;	18:1/22:4;	20:4/20:1;	20:5/20:0				
794.4	[M-H]$^{-}$	PE	40:4	18:0/22:4;	20:2/20:2;	20:4/20:0					
796.4	[M-H]$^{-}$	PE	40:3	18:1/22:2;	20:1/20:2						
798.2	[M-H]$^{-}$	PE	40:2	18:0/22:2;	18:1/22:1;	20:1/20:1;	20:2/20:0				
801.3	[M+CH3COO]$^{-}$	SM	37:2	d18:1/19:1							
802.3	[M+CH3COO]$^{-}$	PC	33:2 or O-34:2								
804.3	[M+CH3COO]$^{-}$	PC	33:1 or O-34:1								
806.4	[M-H]$^{-}$	PE	41:5	19:0/22:5;	21:0/20:5						
808.4	[M-H]$^{-}$	PE	42:11	20:5/22:6							
816.3	[M+CH3COO]$^{-}$	PC	34:2 or O-35:2								
816.4	[M-H]$^{-}$	PE	42:7	20:1/22:6;	20:2/22:5						

m/z	m/z	Phospholipid Class	Molecular species (C:N)	Fatty acyl composition				
818.3	[M+CH3COO]$^-$	PC	34:1					
818.4	[M-H]$^-$	PE	42:6	20:0/22:6;	20:1/22:5;	20:2/22:4;	20:4/22:2	
824.3	[M+CH3COO]$^-$	PC	35:5 or O-36:5					
836.3	[M+CH3COO]$^-$	PC	36:6					
838.3	[M+CH3COO]$^-$	PC	36:5					
844.4	[M+CH3COO]$^-$	PC	36:2					
850.3	[M+CH3COO]$^-$	PC	37:6 or O-38:6					
852.3	[M+CH3COO]$^-$	PC	37:5 or O-38:5					
855.4	[M-H]$^-$	PI	36:5	16:0/20:5;	16:1/20:4			
857.4	[M-H]$^-$	PI	36:4	16:0/20:4;	16:1/20:3			
859.4	[M-H]$^-$	PI	36:3	16:0/20:3;	16:1/20:2;	18:0/18:3;	18:1/18:2	
864.3	[M+CH3COO]$^-$	PC	38:6					
866.3	[M+CH3COO]$^-$	PC	38:5					
869.4	[M-H]$^-$	PI	37:5	17:0/20:5;	17:1/20:4			
876.3	[M+CH3COO]$^-$	PC	38:7					
878.3	[M+CH3COO]$^-$	PC	39:6 or O-40:6					
881.4	[M-H]$^-$	PI	38:6	18:1/20:5;	18:2/20:4			
883.4	[M-H]$^-$	PI	38:5	16:0/22:5;	18:0/20:5;	18:1/20:4		
885.4	[M-H]$^-$	PI	38:4	16:0/22:4;	18:1/20:3;	18:2/20:2		
887.4	[M-H]$^-$	PI	38:3	16:1/22:2;	18:0/20:3;	18:1/20:2		
889.4	[M-H]$^-$	PI	38:2	16:1/22:1;	18:0/20:2;	18:1/20:1;	18:2/20:0	
890.3	[M+CH3COO]$^-$	PC	30:9 or O-40:0 and 40:7					
892.3	[M+CH3COO]$^-$	PC	40:6					
897.5	[M-H]$^-$	PI	39:5 or O-40:5	17:0/22:5;	17:1/22:4;	19:0/20:5;	O-18:0/22:5;	O-18:1/22:4
901.4	[M-H]$^-$	PI	39:3 or O-40:3 and 40:10	17:1/22:2;	19:0/20:3;	20:5/20:5;	O-18:1/22:2	
907.5	[M-H]$^-$	PI	40:7	18:1/22:6;	18:2/22:5;	20:2/20:5;	20:4/20:3	
909.5	[M-H]$^-$	PI	40:6	18:0/22:6;	18:1/22:5			
911.4	[M-H]$^-$	PI	40:5	18:0/22:5;	18:1/22:4;	20:0/20:5;	20:1/20:4;	20:2/20:3
913.4	[M-H]$^-$	PI	40:4	18:0/22:4;	18:2/22:2;	20:0/20:4;	20:1/20:3;	20:2/20:2
1321.6	[M-H]$^-$	CL	62:1					
1337.6	[M-H]$^-$	CL	64:0					
1355.2	[M-H]$^-$	CL	65:1					
1487.6	[M-H]$^-$	CL	74:3					

m/z	m/z	Phospholipid Class	Molecular species (C:N)	Fatty acyl composition
1547.2	$[M-H]^-$	CL	80:14	
1557.1	$[M-H]^-$	CL	80:9	

*12,15-epoxy-13,14-dimethyl-eicosadienoate

Supplementary Table S5.1: Fatty acid (FA) composition (expressed as % of total pool of FAs) identified by GC-MS on *Carcinus maenas* embryos at stage 1 and stage 3. Values are average (± SD) of embryos from three different females (n=3).

Carcinus maenas	**Stage 1**		**Stage3**	
	**Mean**	**SD**	**Mean**	**SD**
C12:0	0.0018	0.0032	0.0000	0.0000
C14:0	0.9546	0.1694	0.6218	0.1858
C15:0	0.8372	0.3694	0.6676	0.2066
C16:0	16.3137	0.3904	18.5953	1.2780
C17:0	0.7944	0.1647	0.9922	0.1467
C18:0	4.2530	0.5350	6.2146	0.3764
C19:0	0.0922	0.0466	0.1066	0.0686
C20:0	0.1450	0.0904	0.1174	0.0636
C22:0	0.0554	0.0203	0.0290	0.0215
**Σ SFA**	**23.4473**	**1.7548**	**27.3444**	**1.2684**
C14:1n5	0.0649	0.0662	0.0282	0.0489
C15:1n1	0.1455	0.0473	0.0486	0.0575
C16:1n5	0.3500	0.0681	0.3912	0.1578
C16:1n7	15.3720	3.2407	10.6776	1.0733
7-methyl-hexadec-6-enoate	0.6405	0.1650	0.4649	0.1176
C17:1n8	0.1257	0.1058	0.0466	0.0808
C17:1n9	0.1110	0.0552	0.1017	0.0583
C18:1n5	0.5127	0.8880	0.0292	0.0506
C18:1n7	4.8435	0.4660	5.8258	0.4798
C18:1n9c	10.8554	1.8536	12.2672	0.5400
C18:1n9t	0.2086	0.0631	0.2062	0.0657
C19:1n8	0.0946	0.1625	0.0184	0.0318
C19:1n9c	0.1868	0.0516	0.0899	0.1358
C20:1n7	2.9278	0.3009	2.4961	0.6690
C20:1n9	1.7158	0.6807	1.2598	0.4449
C22:1n9	0.2092	0.2743	0.0266	0.0233
C22:1n11	0.1454	0.2296	0.0000	0.0000
**Σ MUFA**	**38.5095**	**4.4452**	**33.9780**	**1.0500**
C18:2n3	0.6744	0.2569	0.1908	0.1034
C18:2n6	0.8628	0.3923	0.6536	0.0613
C18:3n3	0.0000	0.0000	0.0000	0.0000
C18:3n3*	1.1166	0.1162	0.7562	0.0679
C19:2n7	0.0777	0.0218	0.0309	0.0351
C20:3n4	0.2682	0.1309	0.3485	0.1010
C20:2n6	0.4334	0.2893	0.0271	0.0470
C20:2n7	0.6329	0.1645	0.7722	0.1259
C20:2n9	0.8645	0.4562	0.9147	0.1629
C22:3n6	0.5003	0.1641	0.2518	0.1388
C22:2n9	0.9436	0.4752	0.3441	0.1632
**Σ PUFA**	**6.3745**	**1.5274**	**4.2899**	**0.4996**
C18:4n3	0.0000	0.0000	0.0000	0.0000
C20:5n3	9.0156	2.0613	14.3223	0.7481
C20:4n6	2.0076	0.3205	2.0455	0.3135
C21:6	0.0393	0.0601	0.0000	0.0000
C21:5n3	0.3621	0.1208	0.1312	0.0818
C22:6n3	9.3595	4.0247	9.6308	2.0175
C22:5n3	2.0572	0.2039	1.3284	0.2404
C22:5n6	0.3221	0.0840	0.0554	0.0870
C22:4n6	0.7832	0.1241	0.7127	0.1425
**Σ HUFA**	**23.9466**	**3.9574**	**28.2263**	**2.7588**

Carcinus maenas	**Stage 1**		**Stage3**	
	**Mean**	**SD**	**Mean**	**SD**
4,8,12-trimethyl-tridecanoate	0.3519	0.2421	0.2758	0.0802
9-methyl-tetradecanoate	0.0000	0.0000	0.0449	0.0777
13-methyl-tetradecanoate (iso)	0.1764	0.0214	0.1984	0.1127
12-methyl-tetradecanoate (anteiso)	0.1384	0.0780	0.1805	0.0804
14-methyl-pentadecanoate (iso)	0.3929	0.1164	0.2715	0.0971
10-Methyl hexadecanoate	0.1014	0.0903	0.1469	0.0338
15-methyl-hexadecanoate (iso)	1.5827	0.1911	1.4026	0.1356
14-methyl-hexadecanoate (anteiso)	1.8503	0.1485	1.5504	0.2375
15-methyl-heptadecanoate (anteiso)	0.0000	0.0000	0.0000	0.0000
16-methyl-heptadecanoate (iso)	0.3576	0.0532	0.3795	0.0433
17-methyl-octadecanoate (iso)	0.0963	0.0385	0.0725	0.0523
16-methyl-octadecanoate (anteiso)	0.4271	0.0981	0.4307	0.1750
**Σ BrFA**	**5.4750**	**0.8166**	**4.9537**	**0.6533**
9,10-methylene-octadecanoate	0.1326	0.0576	0.0741	0.0660
11,12-methylene-octadecanoate	0.0442	0.0766	0.0140	0.0242
11,12-methylene-eicosanoate (1)	0.0624	0.0823	0.0000	0.0000
11,12-methylene-eicosanoate (2)	0.0695	0.0916	0.0285	0.0378
**Σ CyFA**	**0.3087**	**0.2772**	**0.1166**	**0.1200**
10,13-epoxy-11,12-dimethyl-octadecadienoate	0.1939	0.0928	0.0477	0.0538
12,15-epoxy-13,14-dimethyl-eicosadienoate	1.5876	0.2807	0.9994	0.1964
**Σ EpFA**	**1.7814**	**0.2139**	**1.0470**	**0.2427**
F1	0.1570	0.1190	0.0440	0.0394
**Σ UnFA**	**0.1570**	**0.1190**	**0.0440**	**0.0394**
**TOTAL**	**100.0000**		**100.0000**	

* 9c,11t,15c-octadecatrienoate (9,11,15-18:3)

Abbreviations:

SFA – Saturated FA

MUFA – Monounsaturated FA

PUFA – Polyunsaturated FA

HUFA – Highly-polyunsaturated FA

BrFA – Branched FA

CyFA – Cyclic FA

EpFA – Epoxy FA

UnFA – Unknown FA

Supplementary Table S5.2: Fatty acid (FA) composition (expressed as % of total pool of FAs) identified by GC-MS on *Necora puber* embryos at stage 1 and stage 3. Values are average (± SD) of embryos from three different females (n=3).

Necora puber	**Stage 1**		**Stage 3**	
	**Mean**	**SD**	**Mean**	**SD**
C12:0	0.0000		0.0000	
C14:0	1.9321	0.1778	1.4130	0.1845
C15:0	1.2154	0.1761	0.9156	0.0478
C16:0	18.8602	0.3308	19.5742	0.3369
C17:0	0.6671	0.0597	0.8404	0.1721
C18:0	3.4659	0.3148	4.7023	1.0062
C19:0	0.0754	0.0108	0.0741	0.0214
C20:0	0.0874	0.0079	0.0941	0.0215
C22:0	0.0314	0.0544	0.0000	0.0000
**Σ SFA**	**26.3348**	**0.6824**	**27.6137**	**1.2912**
C14:1n5	0.0000		0.0000	
C15:1n1	0.1769	0.0360	0.1198	0.0335
C16:1n5	0.6520	0.1004	0.7784	0.1313
C16:1n7	17.2082	1.1380	12.8234	2.9087
7-methyl-hexadec-6-enoate	0.5824	0.0422	0.5111	0.0748
C17:1n8	0.1336	0.0348	0.1275	0.0190
C17:1n9	0.0000	0.0000	0.0000	0.0000
C18:1n5	0.0000	0.0000	0.0000	0.0000
C18:1n7	4.1246	0.3687	4.9527	0.3056
C18:1n9c	10.6265	0.8736	10.6764	0.4332
C18:1n9t	0.3532	0.0121	0.4668	0.0706
C19:1n8	0.1371	0.0151	0.0983	0.0068
C19:1n9c	0.1134	0.0197	0.1192	0.0431
C20:1n7	2.8793	0.5247	2.8029	0.1651
C20:1n9	1.1618	0.1231	1.1579	0.0803
C22:1n9	0.0000	0.0000	0.0000	0.0000
C22:1n11	0.1111	0.0967	0.0720	0.0628
**Σ MUFA**	**38.2599**	**0.4576**	**34.7063**	**3.4697**
C18:2n3	0.0000		0.0000	
C18:2n6	1.3927	0.0678	0.7847	0.1645
C18:3n3	0.5412	0.0360	0.4167	0.0321
C18:3n3*	1.4856	0.2306	1.0884	0.2682
C19:2n7	0.0000	0.0000	0.0000	0.0000
C20:3n4	0.6056	0.0876	0.8593	0.0230
C20:2n6	1.0140	0.1824	1.0155	0.1283
C20:2n7	0.5436	0.0363	0.6439	0.1743
C20:2n9	1.0318	0.3495	1.0416	0.2075
C22:3n6	0.4469	0.0536	0.4488	0.0371
C22:2n9	1.4200	0.1300	1.1333	0.1426
**Σ PUFA**	**8.4814**	**0.9190**	**7.4321**	**0.5837**
C18:4n3	0.7409	0.0635	0.5231	0.1392
C20:5n3	8.1688	0.5888	10.1091	1.0991
C20:4n6	1.5305	0.1752	2.1904	0.6280
C21:6	0.0000	0.0000	0.0000	0.0000
C21:5n3	0.1551	0.0248	0.1098	0.0246
C22:6n3	7.5820	0.8328	8.0057	1.4822
C22:5n3	1.4824	0.2600	1.7100	0.1071
C22:5n6	0.1510	0.0258	0.1759	0.0573
C22:4n6	0.8003	0.0808	0.9012	0.0470
**Σ HUFA**	**20.6109**	**1.3967**	**23.7252**	**2.1594**

Necora puber	**Stage 1**		**Stage 3**	
	**Mean**	**SD**	**Mean**	**SD**
4,8,12-trimethyl-tridecanoate	0.4653	0.0862	0.2330	0.0837
9-methyl-tetradecanoate	0.0000	0.0000	0.0000	0.0000
13-methyl-tetradecanoate (iso)	0.2339	0.0343	0.2659	0.0993
12-methyl-tetradecanoate (anteiso)	0.1284	0.0937	0.0973	0.0621
14-methyl-pentadecanoate (iso)	0.5466	0.0112	0.4556	0.0146
10-methyl-hexadecanoate	0.2211	0.0445	0.2364	0.0511
15-methyl-hexadecanoate (iso)	1.6652	0.0512	1.6187	0.0605
14-methyl-hexadecanoate (anteiso)	1.9076	0.1141	1.9154	0.2122
15-methyl-heptadecanoate (anteiso)	0.0000	0.0000	0.0000	0.0000
16-methyl-heptadecanoate (iso)	0.0000	0.0000	0.5556	0.1416
17-methyl-octadecanoate (iso)	0.0498	0.0164	0.0482	0.0064
16-methyl-octadecanoate (anteiso)	0.2789	0.0230	0.3338	0.0606
**Σ BrFA**	**5.4969**	**0.0590**	**5.7600**	**0.1961**
9,10-methylene-octadecanoate	0.1091	0.0036	0.1018	0.0128
11,12-methylene-octadecanoate	0.0934	0.0068	0.0746	0.0127
11,12-methylene-eicosanoate (1)	0.0406	0.0052	0.0381	0.0317
11,12-methylene-eicosanoate (2)	0.0000	0.0000	0.0000	0.0000
**Σ CyFA**	**0.2430**	**0.0096**	**0.2146**	**0.0542**
10,13-epoxy-11,12-dimethyl-octadecadienoate	0.0000	0.0000	0.0000	0.0000
12,15-epoxy-13,14-dimethyl-eicosadienoate	0.5351	0.1412	0.5365	0.0890
**Σ EpFA**	**0.5351**	**0.1412**	**0.5365**	**0.0890**
F1	0.0380	0.0119	0.0115	0.0200
**Σ UnFA**	**0.0380**	**0.0119**	**0.0115**	**0.0200**
**Total**	**100.0000**		**100.0000**	

* 9c,11t,15c-octadecatrienoate (9,11,15-18:3)

Abbreviations:

SFA – Saturated FA

MUFA – Monounsaturated FA

PUFA – Polyunsaturated FA

HUFA – Highly-polyunsaturated FA

BrFA – Branched FA

CyFA – Cyclic FA

EpFA – Epoxy FA

UnFA – Unknown FA
